# Reactive oxygen species promote ovarian cancer progression via the HIF-1α/LOX/E-cadherin pathway

**DOI:** 10.3892/or.2014.3448

**Published:** 2014-08-28

**Authors:** YU WANG, JUN MA, HAORAN SHEN, CHENGJIE WANG, YUEPING SUN, STEPHEN B. HOWELL, XINJIAN LIN

**Affiliations:** 1 Department of Obstetrics and Gynecology, Renji Hospital, School of Medicine, Shanghai Jiaotong University, Shanghai, P.R. China; 2 Department of Cell Biology, Key Laboratory of the Education Ministry for Cell Differentiation and Apoptosis, Institutes of Medical Sciences, School of Medicine, Shanghai Jiaotong University, Shanghai, P.R. China; 3 Department of Medicine and UC San Diego Moores Cancer Center, University of California-San Diego, La Jolla, CA, USA

**Keywords:** reactive oxygen species, hypoxia-inducible transcription factor-1, lysyl oxidase, E-cadherin, ovarian carcinomas

## Abstract

Reactive oxygen species (ROS) can drive the de-differentiation of tumor cells leading to the process of epithelial-to-mesenchymal transition (EMT) to enhance invasion and metastasis. The invasive and metastatic phenotype of malignant cells is often linked to loss of E-cadherin expression, a hallmark of EMT. Recent studies have demonstrated that hypoxic exposure causes HIF-1-dependent repression of E-cadherin. However, the mechanism by which ROS and/or HIF suppresses E-cadherin expression remains less clear. In the present study, we found that ROS accumulation in ovarian carcinoma cells upregulated HIF-1α expression and subsequent transcriptional induction of lysyl oxidase (LOX) which repressed E-cadherin. Loss of E-cadherin facilitated ovarian cancer (OC) cell migration *in vitro* and promoted tumor growth *in vivo*. E-cadherin immunoreactivity correlated with International Federation of Gynecology and Obstetrics (FIGO) stage, tumor differentiation and metastasis. Negative E-cadherin expression along with FIGO stage, tumor differentiation and metastasis significantly predicted for a lower 5-year survival rate. These findings suggest that ROS play an important role in the initiation of metastatic growth of OC cells and support a molecular pathway from ROS to aggressive transformation which involves upregulation of HIF-1α and its downstream target LOX to suppress E-cadherin expression leading to an increase in cell motility and invasiveness.

## Introduction

Ovarian cancer (OC) is the fifth leading cause of cancer-related death among women in the USA and has the highest mortality rate of all gynecologic cancers with an estimated 14,030 deaths in 2013 (1). A major contributor to the high mortality rate is the fact that 75% of women with OC present with metastasis at diagnosis (2). Moderate improvement in the 5-year survival rate of OC patients has been observed in recent years, owing to more aggressive debulking surgery and improved chemotherapy regimens including the introduction of taxane/platinum-based chemotherapy, intraperitoneal delivery of chemotherapy, dose-dense chemotherapy and the availability of novel agents such as bevacizumab (3–6). However, the majority of patients still die of their disease, this being mainly attributable to presentation at advanced stage [International Federation of Gynecology and Obstetrics (FIGO) stage III–IV] and to primary or acquired drug resistance (7–9). Since overall survival remains poor, there is an urgent need to further understand the molecular pathways altered in OC that may contribute to the development of metastasis, recurrence and resistance to chemotherapeutic agents.

A majority of solid tumors develop a pathophysiologic microenvironment that is characterized by low oxygen tension (hypoxia) as a result of an inadequate and chaotic blood supply (10,11). Tumor cell populations that adapt to a hypoxic condition tend to undergo transformation into a more aggressive phenotype leading to metastasis and therapy resistance (10,12,13). A key mediator in cellular response to oxygen deprivation is the hypoxia-inducible transcription factor-1 (HIF-1) which comprises a constitutively expressed β-subunit and an oxygen-labile α-subunit. In normoxia, HIF-1α is destabilized by prolyl hydroxylation and targeted for proteasomal degradation. However, under hypoxic conditions where O_2_ is limited for prolyl hydroxylase (PHD) activity, ubiquitination of HIF-1α is inhibited (14). As a result, HIF-1α accumulates and binds to the hypoxia-response elements of various target genes thus activating transcription of these genes that are involved in angiogenesis, energy metabolism, vasomotor regulation, adaptive survival or apoptosis (15,16). Under hypoxia, levels of intracellular reactive oxygen species (ROS) paradoxically increase via the transfer of electrons from ubisemiquinone to molecular oxygen at the Q_0_ site of complex III of the mitochondrial electron transport chain (17). These mitochondrial-derived ROS have been shown to stabilize and activate HIF-1 most likely by modulation of PHD activity (18,19).

Recently, lysyl oxidase (LOX) has been identified as an important regulator of hypoxia-induced tumor progression via an HIF-1α-dependent mechanism in a variety of human cancers including breast, colon, head and neck, ovarian, prostate and renal cell carcinomas (20–24). In fact, LOX is one of the most upregulated genes from a number of gene profiling studies in search for novel HIF-regulated genes (25–27). LOX is a copper-dependent amine oxidase that catalyzes the cross-linking of collagen and elastin in the extracellular matrix (ECM), thereby regulating the tensile strength of tissues (28). LOX has been shown to enhance tumor cell proliferation and invasion and its expression is correlated with poor clinical outcome (20,29,30).

Accumulating evidence indicates that hypoxia can drive the de-differentiation of tumor cells leading to the process of epithelial-to-mesenchymal transition (EMT) to enhance invasion and metastasis (31,32). It is generally conceived that the invasive and metastatic phenotype of malignant cells is associated with downregulation of E-cadherin expression, a hallmark of EMT (33). Recent studies have demonstrated that hypoxic exposure results in HIF-1-dependent repression of E-cadherin; however, the mechanism by which hypoxia and/or HIF suppresses E-cadherin expression remains less clear since a number of different pathways have been suggested (34–36). Intriguingly, in mouse skin carcinoma cell models, LOX has been shown to physically interact with and increase the activity of Snail, a major transcriptional repressor of E-cadherin (37). However, a functional link between LOX and E-cadherin in OC progression in the context of hypoxia has not been described. In the present study, we investigated the role of LOX in hypoxic repression of E-cadherin and report here that ROS lead to activation of HIF-1 and transcriptional induction of LOX, which represses E-cadherin to promote EMT and invasiveness of hypoxic human OC cells.

## Materials and methods

### Cell line cultures

Human ovarian carcinoma SKOV3 cells were purchased from the Chinese Academy of Sciences, Shanghai Cell Bank and maintained in McCoy’s 5A medium containing 10% fetal bovine serum, 100 units/ml penicillin and 100 μg/ml streptomycin in a 5% CO_2_ and 95% air humidified atmosphere at 37°C.

### Patients and tumor samples

Histological samples of 54 patients with epithelial OC were retrieved from the files of the Department of Pathology, Renji Hospital, School of Medicine, Shanghai Jiaotong University. All the tissue samples were obtained from patients aged between 40 and 70 years (mean age, 53 years) who underwent laparotomy at our department from 2005 to 2007. None had received preoperative chemotherapy or radiotherapy. These specimens were fixed in 10% phosphate-buffered formalin and embedded in paraffin. Serial sections (4-μm) were constructed for hematoxylin and eosin staining and for immunohistochemistry. Data including FIGO stage, tumor grade, tumor size at surgery and the presence of lymphovascular invasion were obtained from histopathological reports and patient medical records. According to the FIGO classification, 25 patients were classified as stage I–II, and 29 patients were III–IV. Thirty-eight patients were identified as having moderately-well differentiated tumors and 16 patients had poorly differentiated tumors. Survival time was calculated as the interval from the day of surgery to the last visit or death from an OC-related cause until October 1, 2012. The present study was approved by the Ethics Committee of Shanghai Jiaotong University School of Medicine, and all subjects provided written informed consent.

### Immunohistochemical staining and analysis

At the time of surgery, tumors were dissected and fixed for 24 h in neutral buffered formalin. After fixation, slices were routinely embedded in paraffin wax. Immunostaining for E-cadherin was performed with rabbit polyclonal antibodies against human E-cadherin (clone H-108; Santa Cruz) at a 1:50 dilution. The 4-μm sections were placed on silane-coated slides, deparaffinized and rehydrated in descending concentrations of alcohol, immersed in phosphate-buffered saline (PBS) containing 0.3% hydrogen peroxide and then boiled in 10 mM sodium citrate buffer (pH 6.5) for 15 min in a microwave oven. After blocking with 1% bovine serum albumin in PBS containing 0.05% Tween-20 for 30 min, the slides were incubated with the primary antibody overnight at 4°C. Hematoxylin was used for counterstaining. Negative controls were included by replacing the primary antibody with PBS. E-cadherin expression was categorized on the basis of the intensity of staining, the portion of the circumference of the cytoplasmic membrane stained and the percentage of cells exhibiting membranous staining. Cases with strong complete membranous staining in ≥10% tumor cells were considered positive as used in most studies (38).

### Measurement of ROS production in cells

2,7-Dichlorodihydrofluorescein diacetate (DCFH-DA; Sigma) was used as ROS capture in the cells. It is cleaved intracellularly by non-specific esterases to form 2,7-dichlorodihydrofluorescein (DCFH), which is further oxidized by ROS and becomes a highly fluorescent compound 2,7-dichlorofluorescein (DCF). Therefore, the average fluorescent intensity of DCF is a surrogate measure of intracellular ROS levels. Cultured cells were exposed to various drugs and 10 μM of DCFH-DA at 37°C for 15 min. After washing twice with ice-cold PBS, cells were harvested and immediately subjected to flow cytometry.

### Reverse transcription PCR (RT-PCR)

Total RNA was isolated from cells by TRIzol reagent (Invitrogen, Carlsbad, CA, USA). First-strand cDNA was synthesized using the SuperScript reverse transcriptase (Invitrogen, Germany) and random primers. The paired forward and reverse primers to amplify a specific segment of the cDNA were as follows: 5′-TCCAGCAGACTCAAATACAAGAAC-3′ and 5′-GTATGTGGGTAGGAGATGGAGATG-3′ (for HIF-1α); 5′-GCATACAGGGCAGATGTCAGA-3′ and 5′-GGCATCAAGCAGGTCATAGTG-3′ (for LOX); 5′-TGAAGGTGACAGAGCCTCTGGAT-3′ and 5′-TGGGTGAATTCGGGCTTGTT-3′ (for E-cadherin); and 5′-TGCACCACCAACTGCTTAGC-3′ and 5′-GGCATGGACTGTGGTCATGAG-3′ (for GAPDH). The thermal cycling conditions were: 95°C for 60 sec, 45 cycles of 95°C for 10 sec, 60°C for 15 sec and 72°C for 20 sec. The PCR products were run on 2% agarose gel, and the density of the bands on the gel was quantified by densitometry using the Tocan gel imaging analysis system. Gene expression was presented as the relative yield of the PCR product from the target gene to the reference GAPDH gene. Samples were prepared in triplicate with 3 independent sample sets being analyzed.

### Knockdown with small interfering RNA (siRNA)

For transient knockdown of HIF-1α, SKOV-3 cells were transfected with siRNA oligonucleotides using Lipofectamine 2000 (Invitrogen) according to the manufacturer’s instructions. The sequences of siRNA for HIF-1α and for non-targeting scrambled control were: 5′-CCCUUAUGCACCAACUAGA-3′ and 5′-GCTCAAAGACGCGTTCATA-3′, respectively.

### Western blot analysis

For immunoblot analysis, cells were harvested by scraping, washed in PBS, resuspended, then homogenized and sonicated in RIPA buffer [1X PBS, 1% Nonidet P-40, 0.5% sodium deoxycholate, 0.1% SDS, 1 mM sodium orthovanadate, 10 mg/ml aprotinin and 100 mg/ml phenylmethylsulphonyl fluoride (PMSF)]. Lysates were then centrifuged at 12,000 × g for 10 min at 4°C, and supernatants were collected for analysis. The protein contents in the supernatants were determined by the Bradford reagent assay. The protein was mixed with loading buffer, boiled for 5 min, separated on 7.5% sodium dodecyl sulfate-polyacrylamide (SDS-PAGE) gels at 120 V for 2 h and then transferred to Immunobilon-P polyvinylidene difluoride (PVDF) membranes (Millipore, Bedford, MA, USA). The membranes were blocked with 5% dry milk in PBS and probed with specific antibodies at proper dilutions according to the manufacturer’s instructions and incubated overnight at 4°C. The blots were rinsed and applied with the appropriate secondary antibodies. After washing, the blots were developed using the enhanced chemiluminescence method (ECL; Pierce, Rockford, IL, USA), and the films were scanned using a densitometer. The protein bands were quantified using Quantity One software.

### Wound-healing migration assay

Cells were grown to 90% confluence on 6-well plates, and a scratch was introduced through the cell monolayer using a pipette tip. Baseline (time zero) images were captured, and cell migration was assessed at 48 h by counting the number of cells that had migrated across the scratch and this number was normalized to the scratch area. Data are expressed as the percentage of the control. Cell migration data were obtained from 3 independent wound-healing experiments.

### Nude mouse tumorigenicity

This animal study was approved by the Institutional Animal Care and Use Committee of Renji Hospital, Shanghai Jiaotong University. To assess the effect of ROS on SKOV3 OC cell growth *in vivo*, female nu/nu mice (Charles River Laboratories) were inoculated i.p. with 1×10^6^ SKOV3 cells. Three days after inoculation, the animals were divided randomly into two groups of 10 mice each and treated daily with an i.p. injection of 2.5 g/kg emodin or an equal volume of saline for 3 weeks. Emodin was diluted with sterile 0.9% NaCl to a final volume of 1 ml to allow for adequate peritoneal distribution. Animals were sacrificed 12 weeks after inoculation, and all tumors were removed for measurements of weight and then processed for immunohistochemical staining of E-cadherin expression.

### Statistical analysis

Statistical analysis was performed using SPSS software (version 13.0). Data are presented as means ± standard deviations (SD). Statistical analyses between two groups and among multiple groups were performed with the two-tailed Student’s t-test and one-way analysis of variance (ANOVA), respectively. Chi-square and Fisher’s exact tests were used to evaluate the association between E-cadherin expression and various clinicopathological characteristics. Cox proportional hazard regression model was employed to examine factors of prognostic relevance in univariate analysis of 5-year survival. A P-value <0.05 was considered to indicate a statistically significant result.

## Results

### Expression of HIF-1α, LOX and E-cadherin is influenced by ROS

**Figure 1:**
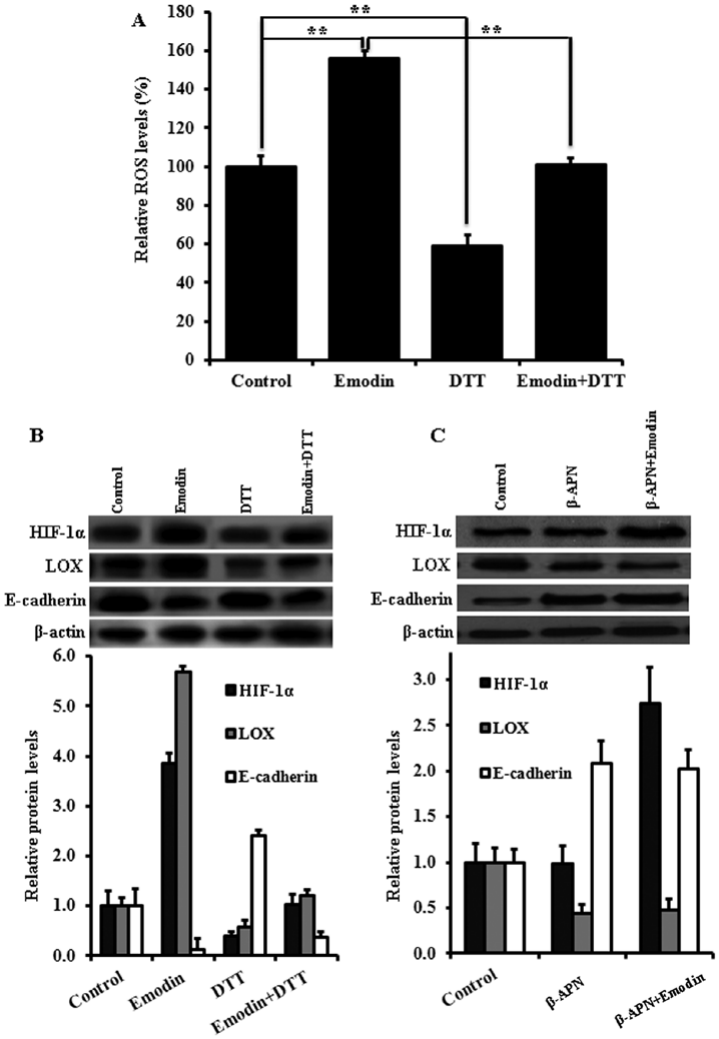
Effect of ROS on HIF-1α, LOX and E-cadherin expression. (A) Assessment of intracellular ROS production in SKOV-3 cells using DCFH-DA method. Cells were treated with 50 μM emodin for 4 h or 5 mM DTT for 2 h or the combination, and the green fluorescent intensity reflecting ROS levels was quantified by flow cytometry. Data are expressed as relative ROS levels to that in the untreated control. Values are means ± SD of 3 independent experiments performed in duplicate. ^**^P<0.01. (B) Representative western blotting showing the expression of HIF-1α, LOX and E-cadherin in SKOV-3 cells after the above indicated treatments; the histogram shows the mean level of the protein determined from 3 independent experiments expressed as the fold-change relative to that in the untreated control cells after normalization to β-actin. (C) Representative western blotting showing the expression of HIF-1α, LOX and E-cadherin in SKOV-3 cells exposed to 100 μM β-APN for 4 h or 100 μM β-APN plus 50 μM emodin for 4 h; the histogram shows the mean level of the protein determined from 3 independent experiments expressed as the fold-change relative to that in the untreated control cells after normalization to β-actin. ROS, reactive oxygen species; LOX, lysyl oxidase; DCFH-DA, 2,7-dichlorodihydrofluorescein diacetate.

Emodin, a natural anthraquinone derivative, was used to generate oxidative stress as it has been shown capable of increasing the production of intracellular ROS (39). As shown in Fig. 1A, the cellular ROS level after exposing SKOV3 cells to emodin was elevated by 55.7% (P<0.01) as compared with the untreated control. Conversely, treatment with DTT, a known ROS scavenger, significantly reduced the ROS level by 41.2% (P<0.01). To confirm a direct role for both emodin and DTT in controlling ROS generation, the effect of the combination of both drugs together on the cellular ROS level was determined. Co-treatment of cells with DTT completely reversed emodin-induced elevation and reduced the cellular ROS to a level equal to that in the untreated control.

To determine whether the differences in ROS accumulation translated into the differential expression of HIF-1α, LOX and E-cadherin, we measured these protein levels by western blot analysis with the specific antibodies (Fig. 1B). Consistent with the ROS data, emodin significantly increased HIF-1α and LOX expression and co-treatment with DTT abolished the induction. In contrast, E-cadherin expression was markedly suppressed in the emodin-treated cells whereas concurrent treatment with DTT restored E-cadherin expression. These results suggest that the increase in HIF-1α and LOX levels that accompanied E-cadherin suppression is attributable to an increase in cellular ROS levels.

To isolate the regulation of E-cadherin by LOX from the ROS-triggered molecular cascade of hypoxia signaling, SKOV3 cells were treated with β-APN, a specific and irreversible inhibitor of LOX enzymatic activity. As shown in Fig. 1C, β-APN treatment alone led to a moderate increase in E-cadherin expression, yet had no effect on the HIF-1α level. However, LOX inhibition did not prevent HIF-1α upregulation by emodin while sustaining the expression of E-cadherin. These data indicate that LOX may serve as an intermediate signaling molecule linking HIF-1α to E-cadherin, the major structural protein of the adherens junction whose suppression is a well-known prerequisite for tumor cell invasion.

### LOX is the transcriptional target of HIF-1α which is inversely correlated with E-cadherin expression

**Figure 2:**
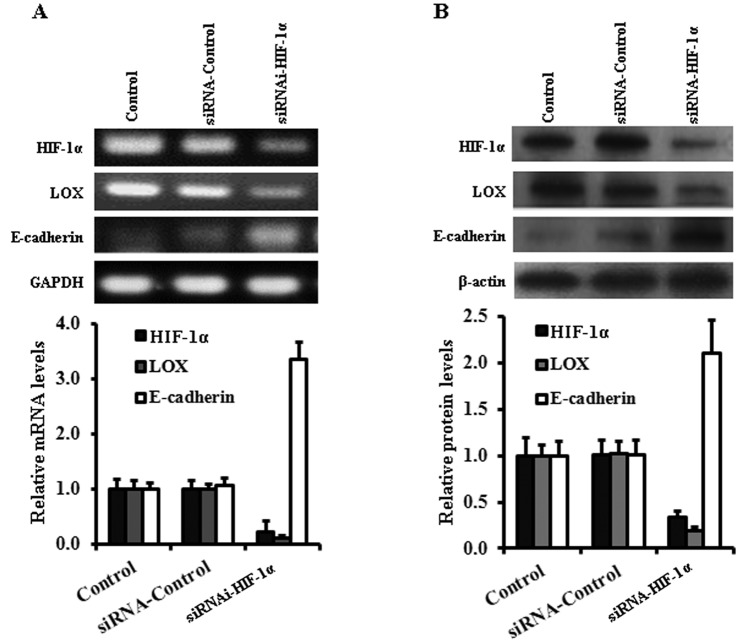
LOX is HIF-1α-dependent and negatively regulates E-cadherin expression. (A) Representative RT-PCR analysis showing the effect of HIF-1α knockdown on LOX and E-cadherin mRNA expression in SKOV-3 cells; the histogram shows the mean level of mRNA determined from 3 independent experiments expressed as the fold-change relative to that in the untreated control cells after normalization to GAPDH. (B) Representative western blotting showing the effect of HIF-1α knockdown on LOX and E-cadherin protein expression in SKOV-3 cells; the histogram shows the mean level of the protein determined from 3 independent experiments expressed as the fold-change relative to that in the untreated control cells after normalization to β-actin. LOX, lysyl oxidase.

To further characterize the HIF-1α-dependent regulation of LOX that mediates E-cadherin expression, HIF-1α was transiently knocked down in SKOV-3 cells. Fig. 2A shows that the SKOV-3 cells transfected with HIF-1α siRNA markedly reduced LOX mRNA expression with a concomitant increase in E-cadherin mRNA levels as compared with the untreated cells or non-targeting scrambled siRNA-transfected cells. Similar changes in the patterns of LOX and E-cadherin protein expression were observed (Fig. 2B). Thus, knockdown of HIF-1α was accompanied by reduced expression of LOX and increased expression of E-cadherin at both the mRNA and protein levels. This suggests that a major component of the effect of HIF-1α on LOX expression is at the transcriptional level and that E-cadherin upregulation consequent to HIF-1α knockdown is possibly through its inhibition of LOX.

### Correlation between E-cadherin expression and clinicopathological features and clinical outcome

**Figure 3:**
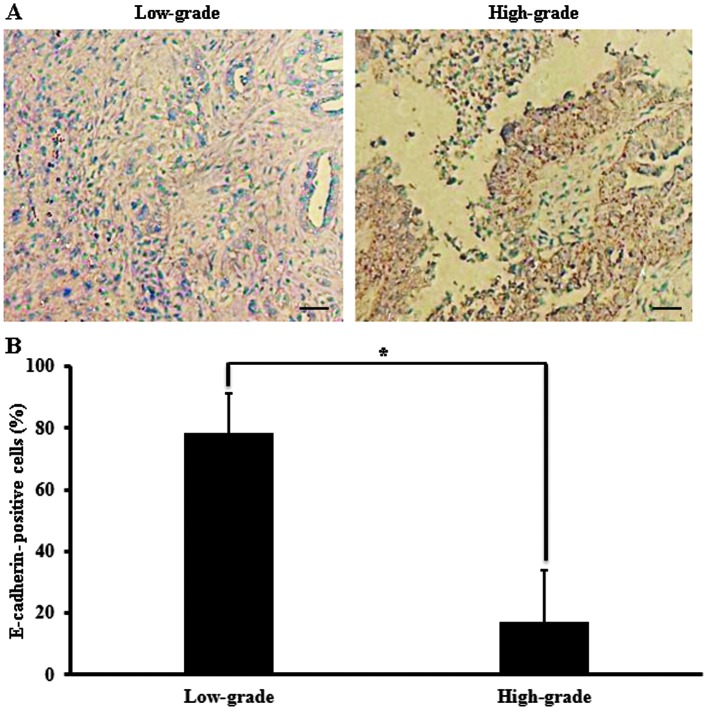
Immunohistochemical staining of E-cadherin in ovarian cancer tissues. (A) Representative images of IHC staining for E-cadherin expression in low- and high-grade tumors. Magnification, ×400. Scale bar, 20 μm. (B) Quantification of E-cadherin-positive cells in the tumors. Five visual fields were randomly selected and 100 cells in each field were counted. Data are presented as the percentage of the total number of positive cells relative to the total 100 cells counted. Values are means ± SD, n=5. ^*^P<0.05.

To validate the aforementioned observations in clinically relevant OC tissues, we examined E-cadherin expression by immunohistochemistry in primary and metastatic human epithelial ovarian carcinoma samples and ascertained the correlation with clinical pathological features and patient survival. Given that our previous study demonstrated a significant correlation between expression of HIF-1α/LOX and tumor grade, size and lymph node metastasis (24), in this study we only focused on the clinical relevance of E-cadherin expression with regard to clinicopathological characteristics and patient survival. Fig. 3 shows sections from representative low- and high-grade OCs stained for E-cadherin in which expression was observed along the cytoplasmic membrane. The correlation between expression of E-cadherin and clinicopathological features of OC patients is summarized in Table I. There was a significant correlation between low expression of E-cadherin and FIGO stage (P=0.030), tumor differentiation (P=0.013) and metastasis (P=0.009), yet no significant correlation with patient age or tumor size was noted (P>0.05). During the 5-year follow-up period, 23 (42.6%) patients of which 20 were FIGO stage III–IV died and 31 (57.4%) of which 23 were FIGO stage I–II survived. Cox multivariate analysis of the 5-year survival rate confirmed that negative E-cadherin expression along with high FIGO stage, poor differentiation and metastasis were associated with a reduced 5-year survival rate (Table II).

### Modulation of cell migratory ability by HIF-1α and LOX under ROS-induced oxidative stress

**Figure 4:**
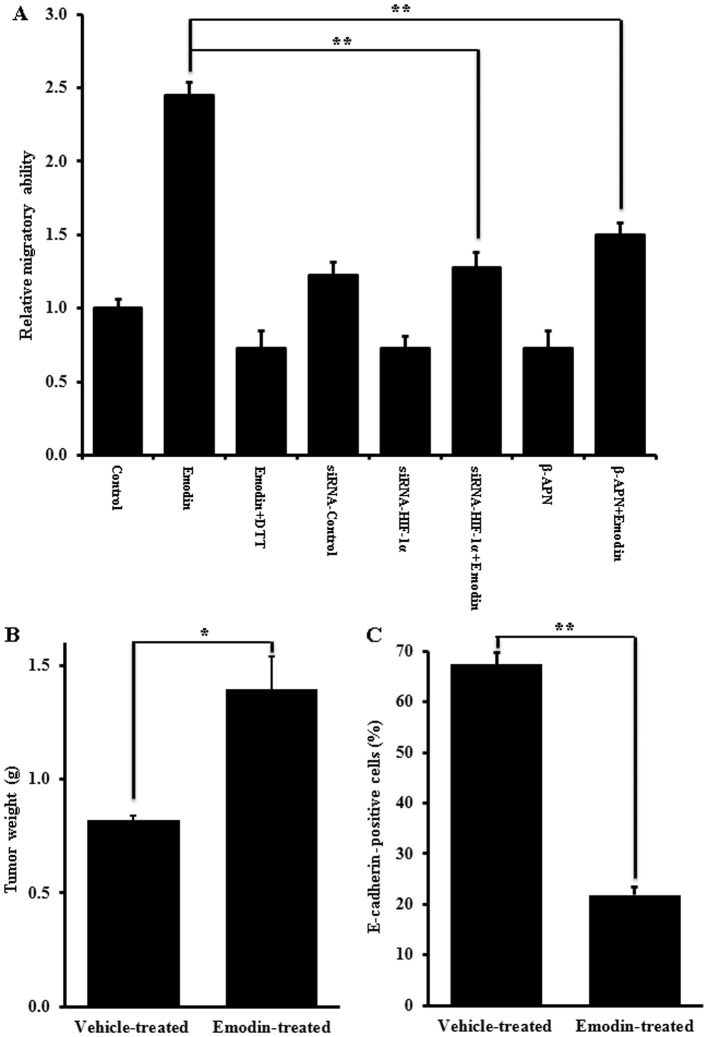
Effect of ROS on SKOV-3 cell migration *in vitro*, tumorigenicity and E-cadherin expression *in vivo*. (A) Relative motility of the SKOV-3 cells under the indicated treatments. Cell migration was assessed by counting the number of cells that had migrated across the scratch. The number of migrated cells was normalized to the scratch area and are expressed as a fold-change relative to the untreated control. Values are means ± SD, n=3. ^**^P<0.01. (B) Tumor weight at the end of 12 weeks after i.p. inoculation of 10^6^ SKOV-3 cells into nude mice. Treatment of the mice commenced on day 3 post-inoculation with an i.p. injection of 2.5 g/kg or saline for 3 weeks. (C) Immunohistochemical quantification of E-cadherin-positive cells in the peritoneal tumors when all the mice were sacrificed 12 weeks after inoculation. ^*^P<0.05, ^**^P<0.01. ROS, reactive oxygen species.

Suppression of E-cadherin expression is regarded as one of the main molecular events responsible for dysfunction in cell-cell adhesion. Given that hypoxic reduction in E-cadherin has been considered as an essential feature of the transitional process shifting from an epithelial cell to a motile and invasive phenotype, we next examined whether ROS stimulated the migration of OC cells and whether LOX plays a role in this effect. The motogenic phenotype was assessed in a wound-healing/scratch assay, a widely accepted method for qualitative assessment of cell migration. The extent of wound closure can be taken as a direct measure of cell motility. As shown in Fig. 4, the migration of SKOV3 cells was strongly induced by emodin. Treatment with ROS scavenger DTT, knockdown of HIF-1α or exposure to the LOX inhibitor β-APN moderately reduced the migration of these cells under normoxic conditions to a similar extent. However, under ROS-induced oxidative stress, HIF-1α knockdown or treatment with LOX β-APN significantly reduced the cell migratory capacity. These results indicate that HIF-1α and LOX, under the influence of ROS, have the ability to affect the migratory potential of cells, a phenomenon that may be of relevance in the metastatic process of OCs.

### ROS promotes tumorigenicity in vivo in association with loss of E-cadherin expression

To assess whether ROS could promote tumor growth *in vivo*, human OC SKOV3 cells were inoculated intraperitoneally in BALB/c nu/nu mice and allowed to form tumor nodules on the peritoneal surface over a period of 12 weeks. As shown in Fig. 4B, the tumor weight in mice that received an i.p. injection of emodin for 21 days was 1.7-fold higher (P<0.05) than that in the vehicle-treated control mice. This result suggests that ROS favor the tumorigenic growth of OC cells. In *in vitro* assays we showed that E-cadherin was suppressed in cells treated with emodin. We confirmed that it was also the case *in vivo*. Fig. 4C shows that a significant reduction in the level of E-cadherin was found in the tumor nodules from the mice treated with emodin. Quantification of the E-cadherin-positive cells revealed that there was a 3.1-fold reduction (P<0.01) of E-cadherin in the emodin-treated tumor nodules compared to the untreated controls. These data provide further support for a role for E-cadherin as a regulator of tumor formation and progression in response to oxidative stress induced by ROS.

## Discussion

Ovarian cancer (OC) is the most lethal gynecological malignancy. Due to deep location of the ovary in the pelvic cavity, usually insidious onset and lack of specific screening programs, the majority of women are diagnosed at the advanced stage of the disease with peritoneal dissemination and distant metastasis. Although aggressive surgical cytoreduction followed by chemotherapy results in complete clinical response in 50–80% of the patients, the overall median survival remains poor (40). Therefore, better understanding of the biology of OC is of clinical relevance, and additional information on molecular and cellular markers may be helpful in predicting tumor progression and response to therapy.

Elevated ROS levels have been observed in many tumors and have emerged as critical signaling stimuli that mediate several important cellular functions in tumor cells including tumorigenesis and metastasis (41). It has been shown that ROS can be produced by the chronic hypoxia of tumor cells, which activate HIF-1α and downstream pathways, enabling tumor cells to acquire invasive competence (42,43). In the present study, we showed that the expression levels of E-cadherin mRNA and protein were substantially reduced when HIF-1α was induced by increased intracellular ROS levels, and that this effect was reversible when HIF-1α was knocked down by an siRNA targeted to HIF-1α. The molecular mechanism of E-cadherin suppression is not clear; however, lysyl oxidase (LOX) has been implicated in the regulation of E-cadherin (22). LOX is a copper-dependent amine oxidase whose expression in the extracellular matrix is closely correlated with tumor development, progression, adhesion, malignant transformation and invasion (44). Kirschmann *et al* found that, compared with poorly invasive/metastatic breast cancer cell line MCF-7, LOX showed higher expression in the highly invasive/metastatic breast cancer MDA-MB-231 cell line (42). In addition, treatment of MDA-MB-231 cells with β-APN decreased invasive activity. Our observation that expression of LOX was significantly increased upon HIF-1α induction and completely abolished with HIF-1α knockdown suggests that LOX may be a direct target of HIF-1α. Intriguingly, irreversible inhibition of LOX activity by β-APN did not prevent ROS-induced HIF-1α upregulation but blocked hypoxic repression of E-cadherin. These results are in good agreement with a previous study (22) indicating that under hypoxic conditions LOX is an intermediate signaling molecule linking HIF-1α to adherens junction molecule E-cadherin whose reduction is a characteristic feature of cells that have gone through an epithelial-to-mesenchymal transition and widely believed to amplify tumor invasiveness and progression. In the present study, we used emodin to enforce the excessive generation of ROS in OC cells and found that such overproduction of ROS repressed E-cadherin expression which was associated with increased migratory capacity of the cells *in vitro*. More importantly, scavenging ROS by DTT, knockdown of HIF-1α by HIF-1α-specific siRNA or inhibition of LOX by β-APN reduced cell migration equally well. In the animal experiment, our results highlighted that ROS promoted OC cell proliferation and tumor formation with concomitant loss of E-cadherin. In keeping with those findings, combined use of an ROS scavenger, HIF inhibitor and LOX-targeted drug may be a productive and efficient way to improve cancer therapy for metastatic disease.

Analysis of the correlation between E-cadherin expression in 54 OC patient tissues and clinicopathological features and patient survival demonstrated that E-cadherin immunoreactivity was associated with FIGO stage, tumor differentiation and the presence of metastasis; the 5-year survival rate of patients with negative E-cadherin expression was significantly lower than the survival rate of patients with positive-E-cadherin expression. Lower expression of E-cadherin was observed in high-grade (FIGO stage III–IV) and poorly differentiated tumors, and correlated with poor survival. However, no significant relationship was found between the 5-year survival rate and patient age or tumor size. While a larger cohort should be tested in a prospective study to assess the precise clinical relevance of E-cadherin expression in ovarian cancers, the results from this study indicate that E-cadherin may serve as a critical marker not only for prediction of the prognosis of OC patients yet also for selection of patients who are at high risk of suffering an unfavorable clinical outcome and thus require more aggressive therapeutic modalities.

In conclusion, the present study demonstrated that ROS promote the migration and metastatic growth of OC cells via upregulation of HIF-1α and LOX and E-cadherin repression. Therefore, ROS itself and the HIF-1α signaling pathways may present potential targets to be exploited therapeutically in patients with metastatic and recurrent ovarian cancers.

## Figures and Tables

**Table I:** Correlation between E-cadherin expression and clinicopathological features.

		E-cadherin		
			
Parameter	n	Positive	Negative	P-value
Age (years)	54	28	26	>0.05
<50	26	12	14	
≥50	28	16	12	
Tumor size (cm)				>0.05
≤2	22	13	9	
>2	32	15	17	
FIGO stage				0.030
I–II	25	19	6	
III–IV	29	9	20	
Tumor grade				0.013
Moderate to well	38	25	13	
Poor	16	3	13	
Metastasis				0.009
Positive	33	10	23	
Negative	21	18	3	

Cases with strong complete membranous staining in ≥10% tumor cells were considered positive. FIGO, International Federation of Gynecology and Obstetrics.

**Table II:** Multivariate analysis of the effect of E-cadherin expression, FIGO stage and tumor differentiation.

Covariate	HR	95% CI	P-value
E-cadherin	2.92	1.52–3.24	P<0.05
Positive			
Negative			
FIGO stage	1.51	1.22–2.53	P<0.05
I–II			
III–IV			
Differentiation	2.25	1.38–2.74	P<0.05
Moderate-well			
Poor			
Metastasis	2.94	1.36–3.68	P<0.05
No			
Yes			

FIGO, International Federation of Gynecology and Obstetrics; HR, hazard risk; CI, confidence interval.
